# *tert*-Butyl [(4-fluoro-3-isopropoxyisoxazol-5-yl)meth­yl](phenyl­sulfon­yl)carbamate

**DOI:** 10.1107/S2414314625003852

**Published:** 2025-05-02

**Authors:** Mohd Abdul Fatah Abdul Manan, David B. Cordes

**Affiliations:** a Faculty of Applied Sciences, Universiti Teknologi MARA, 40450 Shah Alam, Selangor, Malaysia; b EaStCHEM School of Chemistry, University of St Andrews, St Andrews, Fife KY16 9ST, United Kingdom; Katholieke Universiteit Leuven, Belgium

**Keywords:** crystal structure, isoxazole, fluorine, *N*-fluoro­benzene­sulfonimide, sulfonamide, bifurcated hydrogen bond

## Abstract

The title compound, C_18_H_23_FN_2_O_6_S, a new derivative of a fluoro­isoxazole containing sulfonamide functionality has been structurally characterized. The C—S—N—C_ipr_ and C—S—N—C_carb_ (ipr = 3-isopropoxyisoxazole, carb = carbamate) torsion angles are 111.1 (3)° and −70.0 (4)°, respectively. The sulfonamide functional group of this structure features S=O bond lengths of 1.403 (3) and 1.433 (3) Å, an S—N bond length of 1.672 (4) Å, and an S—C bond length of 1.753 (4) Å. The crystal packing features non-classical C—H⋯O hydrogen-bond inter­actions, with the carbonyl atom acting as a bifurcated acceptor, resulting in an *R*^1^_2_(8) ring.

## Abstract 2

The crystal structure of a 4-fluoro­isoxazole compound bearing a sulfonamide functionality is described.

## Graphical abstract

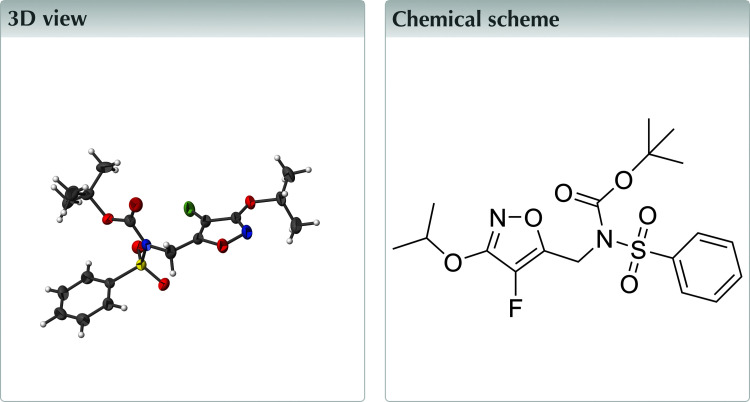

## Structure description

Isoxazoles carrying sulfonamide moieties are very important structural motifs and have gained inter­est from pharmaceutical industry and medicinal chemists owing to their various bioactivities; anti­bacterial (Esfahani *et al.*, 2021 ▸; Martinez *et al.*, 2025 ▸), anti­fungal (Soliman *et al.*, 2025 ▸), anti­cancer (Kilbile *et al.*, 2024 ▸; Vaickelionienė *et al.*, 2023 ▸), anti-inflammatory, anti­diabetic and anti­oxidant (Ahmad *et al.*, 2023 ▸; Dayma *et al.*, 2020 ▸). Pharmaceutically important examples of isoxazole-containing sulfonamide drugs include the anti­bacterial agents sulfisoxazole and sulfamethoxazole (Rusu *et al.*, 2023 ▸), and the anti­obesity and anti­convulsant agent zonisamide (Gidal *et al.*, 2024 ▸). Despite the potential usage of fluorinated five-membered heterocyclic compounds and their functionalization in the life science industries (Imberg *et al.*, 2025 ▸; Hawk *et al.*, 2021 ▸; Fuchibe *et al.*, 2023 ▸), studies pertaining to the synthesis of such structural units, particularly selective fluorination of five-membered isoxazole systems are rare and challenging. To address this limitation, we report herein the crystal structure of the title compound **1**, obtained by treatment of **2** with excess *N*-fluoro­benzene­sulfonimide.

**Figure 1:**
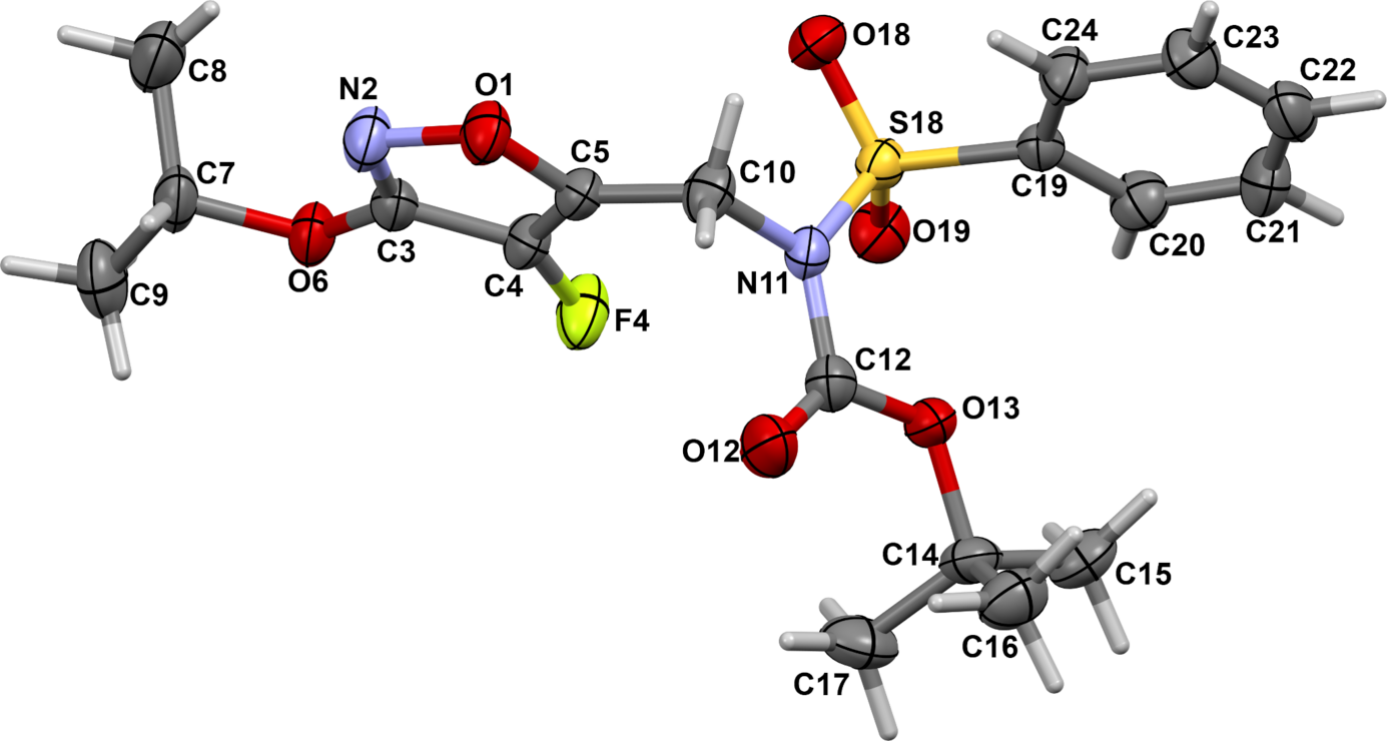
The mol­ecular structure of the title compound, showing displacement ellipsoids drawn at the 50% probability level.

**Figure 2:**
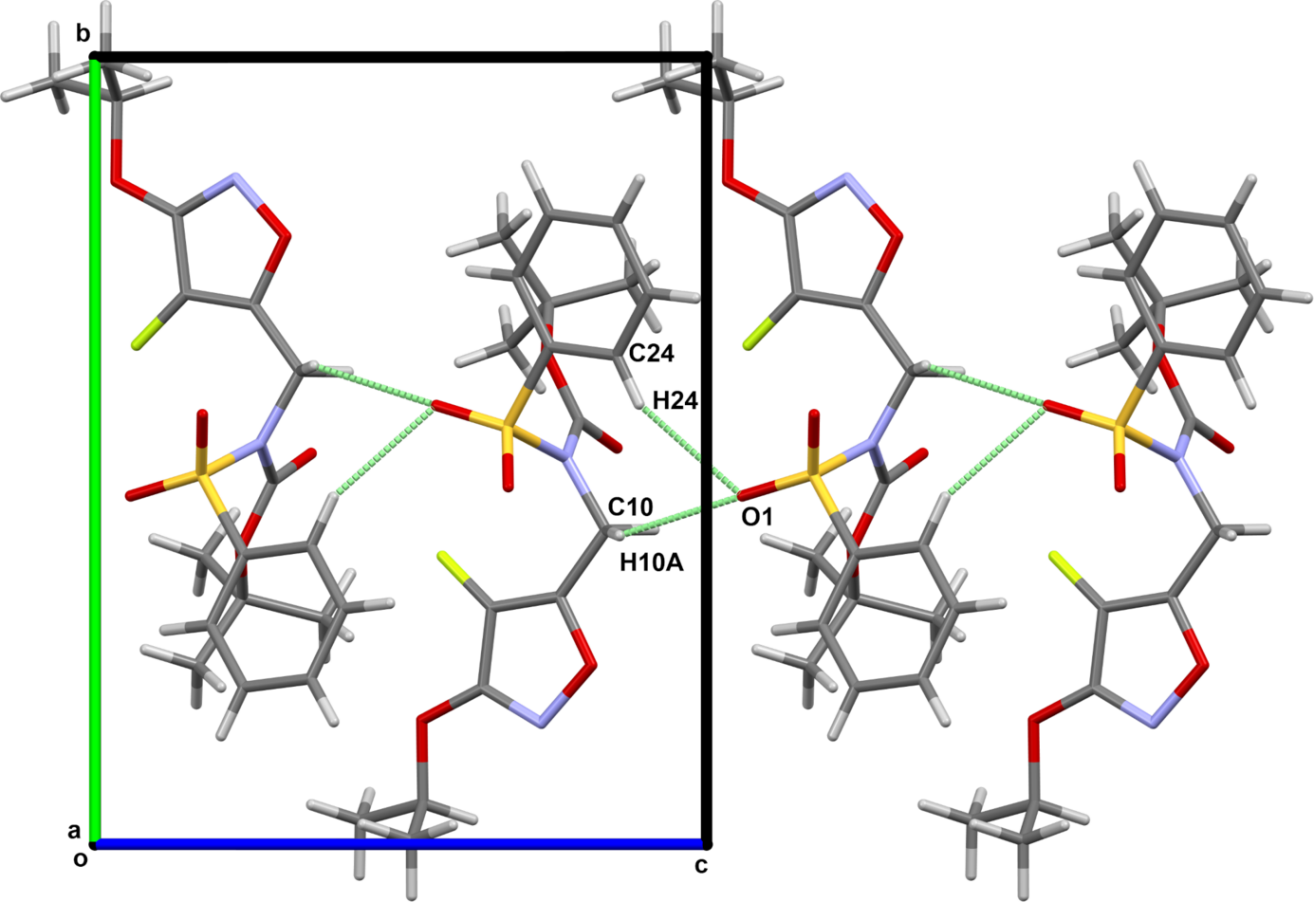
View of the weakly hydrogen-bonded 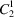
(5) chains.

**Figure 3:**
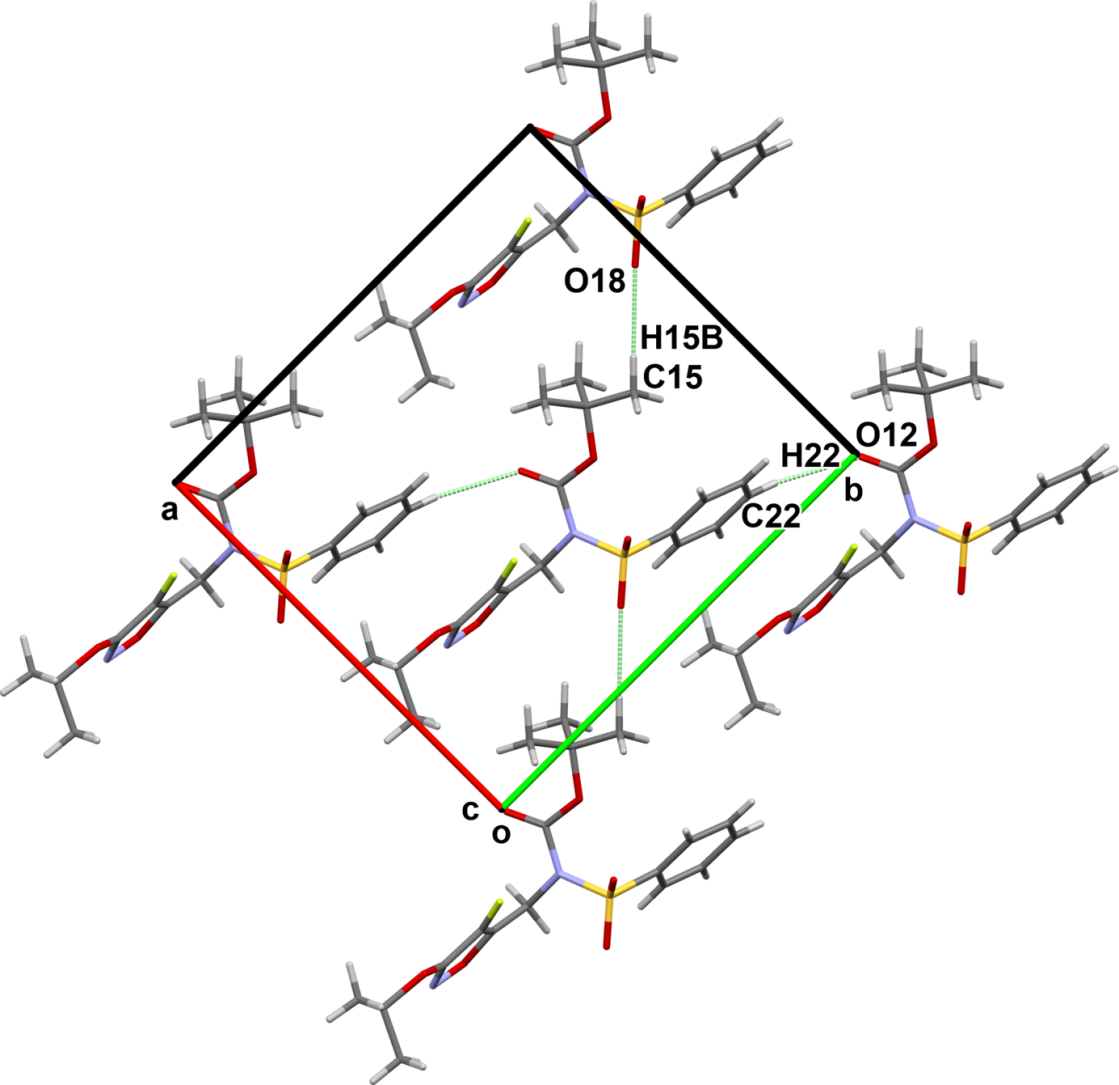
View of the weakly hydrogen-bonded *C*(8) (vertical) and *C*(9) (horizontal) chains.

The mol­ecular structure of the title compound, **1**, which consists of a 4-fluoro­isoxazole derivative with a sulfonamide group is shown in Fig. 1 ▸. In the solid state, the isoxazole ring (O1/N2/C3–C5) forms a dihedral angle of 10.9 (3)° with the sulfonyl-bound phenyl ring (C19–C24). The torsion angles C19—S18—N11—C10 and C19—S18—N11—C12 are 111.2 (3)° and −70.0 (4)° respectively. The sulfonamide adopts a conformation in agreement with that seen in related structures (Khrustalev *et al.*, 2022 ▸; Madhan *et al.*, 2024 ▸; Moroni *et al.*, 2024 ▸). The nitro­gen atom of the sulfonamide displays a *sp^2^* character, with an S18—N11—C10 angle of 119.1 (3)°. The sulfonamide sulfur atom displays a distorted tetra­hedral geometry, with the widening of the O18—S18—O19 angle of 119.2 (2)°, accompanied by simultaneous decrease in the N11—S18—C19 angle [106.0 (2)°], as typically found in *R*SO_2_N*R′* sulfonamide systems (Hernández *et al.*, 2017 ▸; Moroni *et al.*, 2024 ▸). The C10—N11—C12—O12 fragment adopts a *syn* conformation with a torsion angle of 5.0 (6)°. The mol­ecular packing features weak C—H⋯O hydrogen bonds (Table 1 ▸). Atoms C10 and C24 act as donors to the double-acceptor O-atom, O19, enclosing 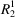
(8) ring motifs, and resulting in the formation of 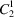
(5) chains along [001] (Fig. 2 ▸). Further chains are formed by other C—H⋯O hydrogen bonds; C15—H15*B* with O18 forming *C*(8) chains along [110], and C22—H22 with O12 forming *C*(9) chains along [1
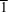
0] (Fig. 3 ▸). The combination of these bonds results in a weakly inter­acting three-dimensional network.

## Synthesis and crystallization

**Figure 4:**
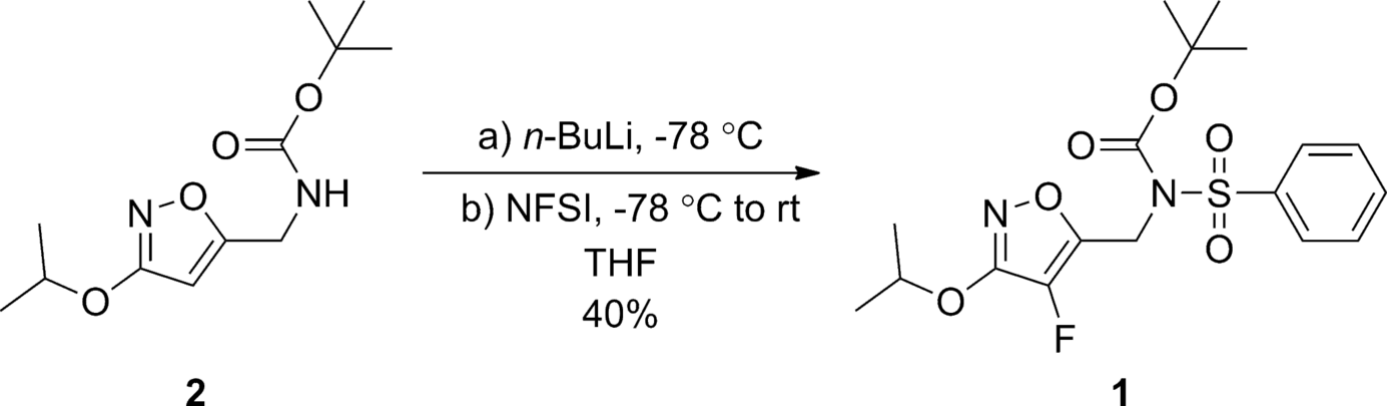
A synthetic scheme for the preparation of the title compound.

The carbamate precursor **2**, was prepared according to our previously established protocol (Abdul Manan *et al.*, 2017 ▸). The title compound **1**, was synthesized following a literature procedure with a minor modification (Abdul Manan *et al.*, 2017 ▸) (Fig. 4 ▸). *n*-BuLi (1.7 ml, 2.5 *M* in hexane, 4.29 mmol, 2.2 eq) was added dropwise to a solution of 5-(*tert*-butyl­oxycarbon­yl)amino­methyl-3-isopropoxyisoxazole, **2**, (500 mg, 1.95 mmol, 1.0 eq) at 195 K. The mixture was stirred for 1.5 h at 195 K and a solution of *N*-fluoro­benzene­sulfonimide (NFSI) (1.23 g, 3.90 mmol, 2.0 eq) in THF (4 ml) was added. The mixture was stirred for 2 h at 195 K and the temperature was allowed to warm to room temperature over 12 h. The reaction mixture was quenched with aqueous NH_4_Cl (10 ml) and the organic phase was extracted into EtOAc (3 × 20 ml). The combined organic layers were dried over MgSO_4_, filtered and concentrated under reduced pressure. The resulting residue was purified by silica gel column chromatography (petroleum ether/Et_2_O, 80:20) to yield the title compound (323 mg, 40%) as a colourless viscous oil that crystallized on standing.

## Refinement

Crystal data, data collection and structure refinement details are summarized in Table 2 ▸.

## Supplementary Material

Crystal structure: contains datablock(s) I. DOI: 10.1107/S2414314625003852/vm4067sup1.cif

Structure factors: contains datablock(s) I. DOI: 10.1107/S2414314625003852/vm4067Isup2.hkl

Supporting information file. DOI: 10.1107/S2414314625003852/vm4067Isup3.cml

CCDC reference: 2447656

Additional supporting information:  crystallographic information; 3D view; checkCIF report

## Figures and Tables

**Table 1:** Hydrogen-bond geometry (Å, °)

*D*—H⋯*A*	*D*—H	H⋯*A*	*D*⋯*A*	*D*—H⋯*A*
C10—H10*A*⋯O19^i^	0.99	2.47	3.121 (6)	123
C15—H15*B*⋯O18^ii^	0.98	2.59	3.571 (6)	176
C22—H22⋯O12^iii^	0.95	2.51	3.335 (6)	145
C24—H24⋯O19^i^	0.95	2.51	3.366 (5)	150

Symmetry codes: (i) 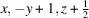; (ii) 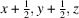; (iii) 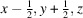.

**Table 2:** Experimental details

Crystal data	
Chemical formula	C_18_H_23_FN_2_O_6_S
*M*_r_	414.44
Crystal system, space group	Monoclinic, *Cc*
Temperature (K)	173
*a*, *b*, *c* (Å)	13.271 (3), 13.904 (3), 11.206 (2)
β (°)	105.547 (6)
*V* (Å^3^)	1992.1 (7)
*Z*	4
Radiation type	Mo *K*α
μ (mm^−1^)	0.21
Crystal size (mm)	0.11 × 0.04 × 0.02

Data collection	
Diffractometer	Rigaku XtaLAB P200K
Absorption correction	Multi-scan (*REQAB*; Rigaku, 1998 ▸)
*T*_min_, *T*_max_	0.828, 0.996
No. of measured, independent and observed [*I* > 2σ(*I*)] reflections	11991, 3548, 2824
*R*_int_	0.060
(sin θ/λ)_max_ (Å^−1^)	0.603

Refinement	
*R*[*F*^2^ > 2σ(*F*^2^)], *wR*(*F*^2^), *S*	0.042, 0.090, 1.02
No. of reflections	3548
No. of parameters	258
No. of restraints	2
H-atom treatment	H-atom parameters constrained
Δρ_max_, Δρ_min_ (e Å^−3^)	0.19, −0.21
Absolute structure	Flack *x* determined using 1114 quotients [(*I*^+^)−(*I*^−^)]/[(*I*^+^)+(*I*^−^)] (Parsons *et al.*, 2013 ▸)
Absolute structure parameter	−0.08 (6)

Computer programs: *CrystalClear-SM Expert* (Rigaku, 2015 ▸), *SUPERFLIP* (Palatinus & Chapuis, 2007 ▸), *SHELXL2019/3* (Sheldrick, 2015 ▸), *Mercury* (Macrae *et al.*, 2020 ▸), *OLEX2* (Dolomanov *et al.*, 2009 ▸) and *publCIF* (Westrip, 2010 ▸).

## full crystallographic data

### tert-Butyl [(4-fluoro-3-isopropoxyisoxazol-5-yl)methyl](phenylsulfonyl)carbamate Crystal data

**Table:**

C_18_H_23_FN_2_O_6_S	*F*(000) = 872
*M**_r_* = 414.44	*D*_x_ = 1.382 Mg m^−^^3^
Monoclinic, *C**c*	Mo *K*α radiation, λ = 0.71073 Å
*a* = 13.271 (3) Å	Cell parameters from 2002 reflections
*b* = 13.904 (3) Å	θ = 2.2–25.3°
*c* = 11.206 (2) Å	µ = 0.21 mm^−^^1^
β = 105.547 (6)°	*T* = 173 K
*V* = 1992.1 (7) Å^3^	Chip, colorless
*Z* = 4	0.11 × 0.04 × 0.02 mm

### tert-Butyl [(4-fluoro-3-isopropoxyisoxazol-5-yl)methyl](phenylsulfonyl)carbamate Data collection

**Table:**

Rigaku XtaLAB P200K diffractometer	3548 independent reflections
Radiation source: Rotating Anode, Rigaku FR-X	2824 reflections with *I* > 2σ(*I*)
Rigaku Osmic Confocal Optical System monochromator	*R*_int_ = 0.060
Detector resolution: 5.8140 pixels mm^-1^	θ_max_ = 25.4°, θ_min_ = 2.2°
shutterless scans	*h* = −16→15
Absorption correction: multi-scan (*REQAB*; Rigaku, 1998)	*k* = −16→16
*T*_min_ = 0.828, *T*_max_ = 0.996	*l* = −13→13
11991 measured reflections	

### tert-Butyl [(4-fluoro-3-isopropoxyisoxazol-5-yl)methyl](phenylsulfonyl)carbamate Refinement

**Table:**

Refinement on *F*^2^	Hydrogen site location: inferred from neighbouring sites
Least-squares matrix: full	H-atom parameters constrained
*R*[*F*^2^ > 2σ(*F*^2^)] = 0.042	*w* = 1/[σ^2^(*F*_o_^2^) + (0.0422*P*)^2^ + 0.1932*P*] where *P* = (*F*_o_^2^ + 2*F*_c_^2^)/3
*wR*(*F*^2^) = 0.090	(Δ/σ)_max_ < 0.001
*S* = 1.01	Δρ_max_ = 0.19 e Å^−^^3^
3548 reflections	Δρ_min_ = −0.21 e Å^−^^3^
258 parameters	Absolute structure: Flack *x* determined using 1114 quotients [(*I*^+^)-(*I*^-^)]/[(*I*^+^)+(*I*^-^)] (Parsons *et al.*, 2013)
2 restraints	Absolute structure parameter: −0.08 (6)
Primary atom site location: iterative	

### tert-Butyl [(4-fluoro-3-isopropoxyisoxazol-5-yl)methyl](phenylsulfonyl)carbamate Special details

**Table:**

Geometry. All esds (except the esd in the dihedral angle between two l.s. planes) are estimated using the full covariance matrix. The cell esds are taken into account individually in the estimation of esds in distances, angles and torsion angles; correlations between esds in cell parameters are only used when they are defined by crystal symmetry. An approximate (isotropic) treatment of cell esds is used for estimating esds involving l.s. planes.
Refinement. The C-bound H atoms were located geometrically (phenyl C—H = 0.95 Å, methine C—H = 1.00 Å, methylene C—H = 0.99 Å, methyl C—H = 0.98 Å) and refined as riding atoms. The constraint *U*_iso_(H) = 1.2*U*_eq_(non-methyl C) or 1.5*U*_eq_(methyl C) was applied in all cases.

### tert-Butyl [(4-fluoro-3-isopropoxyisoxazol-5-yl)methyl](phenylsulfonyl)carbamate Fractional atomic coordinates and isotropic or equivalent isotropic displacement parameters (Å^2^)

**Table:**

	*x*	*y*	*z*	*U*_iso_*/*U*_eq_	
S18	0.20066 (8)	0.53009 (8)	0.67391 (9)	0.0319 (3)	
F4	0.3603 (2)	0.36593 (18)	0.5679 (3)	0.0508 (8)	
O1	0.3211 (2)	0.2277 (2)	0.8123 (3)	0.0405 (8)	
O6	0.3663 (3)	0.1578 (2)	0.5344 (3)	0.0394 (8)	
O12	0.4876 (3)	0.5034 (2)	0.8535 (4)	0.0509 (9)	
O13	0.3957 (2)	0.6237 (2)	0.7380 (3)	0.0374 (8)	
O18	0.1283 (2)	0.4542 (2)	0.6758 (3)	0.0423 (8)	
O19	0.2191 (2)	0.5575 (2)	0.5583 (3)	0.0410 (8)	
N2	0.3347 (3)	0.1538 (3)	0.7299 (4)	0.0424 (10)	
N11	0.3121 (3)	0.4906 (2)	0.7701 (3)	0.0329 (9)	
C3	0.3498 (3)	0.1989 (3)	0.6341 (5)	0.0334 (11)	
C4	0.3455 (4)	0.3008 (3)	0.6489 (4)	0.0340 (11)	
C5	0.3268 (3)	0.3148 (3)	0.7593 (4)	0.0328 (10)	
C7	0.3772 (4)	0.0519 (3)	0.5362 (5)	0.0426 (12)	
H7	0.424446	0.031201	0.617519	0.051*	
C8	0.2719 (4)	0.0050 (4)	0.5175 (6)	0.0623 (17)	
H8A	0.241505	0.024101	0.584538	0.094*	
H8B	0.280006	−0.065051	0.517834	0.094*	
H8C	0.225618	0.025455	0.437850	0.094*	
C9	0.4282 (4)	0.0302 (4)	0.4340 (5)	0.0558 (15)	
H9A	0.381604	0.050097	0.354336	0.084*	
H9B	0.441762	−0.038986	0.432310	0.084*	
H9C	0.494386	0.065447	0.449076	0.084*	
C10	0.3119 (4)	0.3991 (3)	0.8359 (4)	0.0374 (11)	
H10A	0.244691	0.392111	0.857520	0.045*	
H10B	0.368739	0.399875	0.913979	0.045*	
C12	0.4078 (4)	0.5381 (3)	0.7916 (4)	0.0356 (11)	
C14	0.4874 (4)	0.6868 (4)	0.7417 (5)	0.0398 (12)	
C15	0.4359 (4)	0.7729 (4)	0.6674 (5)	0.0501 (14)	
H15A	0.401071	0.752770	0.582521	0.075*	
H15B	0.489062	0.821429	0.665758	0.075*	
H15C	0.384091	0.800369	0.705706	0.075*	
C16	0.5409 (4)	0.7130 (4)	0.8742 (5)	0.0523 (14)	
H16A	0.488817	0.736331	0.914923	0.078*	
H16B	0.592661	0.763617	0.875697	0.078*	
H16C	0.576024	0.656181	0.918007	0.078*	
C17	0.5602 (4)	0.6343 (4)	0.6781 (5)	0.0547 (15)	
H17A	0.591901	0.579010	0.728503	0.082*	
H17B	0.615281	0.678349	0.668906	0.082*	
H17C	0.519972	0.612039	0.596216	0.082*	
C19	0.1648 (3)	0.6322 (3)	0.7446 (4)	0.0283 (10)	
C20	0.1626 (4)	0.7214 (3)	0.6888 (4)	0.0405 (12)	
H20	0.183187	0.728238	0.614288	0.049*	
C21	0.1299 (4)	0.8004 (3)	0.7437 (5)	0.0497 (14)	
H21	0.127115	0.861996	0.706421	0.060*	
C22	0.1013 (4)	0.7896 (4)	0.8526 (5)	0.0452 (13)	
H22	0.079839	0.844194	0.890452	0.054*	
C23	0.1037 (4)	0.7005 (4)	0.9071 (5)	0.0439 (12)	
H23	0.083502	0.693720	0.981837	0.053*	
C24	0.1353 (4)	0.6210 (3)	0.8528 (4)	0.0391 (12)	
H24	0.136771	0.559284	0.889508	0.047*	

### tert-Butyl [(4-fluoro-3-isopropoxyisoxazol-5-yl)methyl](phenylsulfonyl)carbamate Atomic displacement parameters (Å^2^)

**Table:**

	*U*^11^	*U*^22^	*U*^33^	*U*^12^	*U*^13^	*U*^23^
S18	0.0352 (6)	0.0284 (5)	0.0340 (6)	−0.0033 (5)	0.0127 (5)	−0.0042 (5)
F4	0.083 (2)	0.0287 (14)	0.0528 (18)	0.0026 (14)	0.0383 (16)	0.0038 (13)
O1	0.054 (2)	0.0259 (17)	0.0449 (19)	0.0021 (15)	0.0193 (16)	0.0043 (14)
O6	0.049 (2)	0.0253 (16)	0.045 (2)	0.0029 (14)	0.0144 (17)	−0.0042 (14)
O12	0.040 (2)	0.0382 (19)	0.071 (2)	0.0057 (17)	0.0076 (18)	0.0059 (18)
O13	0.0303 (17)	0.0292 (17)	0.054 (2)	−0.0019 (13)	0.0133 (15)	0.0036 (15)
O18	0.042 (2)	0.0334 (18)	0.053 (2)	−0.0099 (15)	0.0160 (16)	−0.0045 (16)
O19	0.051 (2)	0.0420 (18)	0.0326 (18)	−0.0035 (15)	0.0152 (15)	−0.0024 (14)
N2	0.055 (3)	0.025 (2)	0.051 (3)	0.0008 (18)	0.020 (2)	−0.0018 (19)
N11	0.037 (2)	0.0223 (19)	0.042 (2)	0.0015 (16)	0.0153 (18)	−0.0014 (16)
C3	0.031 (2)	0.025 (2)	0.044 (3)	0.0009 (19)	0.009 (2)	−0.001 (2)
C4	0.039 (3)	0.023 (2)	0.041 (3)	0.001 (2)	0.012 (2)	0.006 (2)
C5	0.033 (3)	0.024 (2)	0.042 (3)	−0.0009 (19)	0.012 (2)	0.002 (2)
C7	0.044 (3)	0.024 (2)	0.056 (3)	0.007 (2)	0.006 (3)	−0.005 (2)
C8	0.056 (3)	0.033 (3)	0.093 (5)	−0.001 (3)	0.010 (3)	−0.009 (3)
C9	0.067 (4)	0.042 (3)	0.059 (4)	0.013 (3)	0.019 (3)	−0.014 (3)
C10	0.051 (3)	0.029 (2)	0.034 (3)	−0.002 (2)	0.014 (2)	0.002 (2)
C12	0.036 (3)	0.031 (3)	0.040 (3)	0.002 (2)	0.011 (2)	−0.003 (2)
C14	0.030 (2)	0.042 (3)	0.049 (3)	−0.011 (2)	0.012 (2)	−0.005 (2)
C15	0.054 (4)	0.040 (3)	0.057 (3)	−0.014 (2)	0.018 (3)	0.005 (2)
C16	0.054 (3)	0.051 (3)	0.052 (3)	−0.016 (3)	0.015 (3)	−0.009 (3)
C17	0.039 (3)	0.074 (4)	0.058 (4)	−0.006 (3)	0.025 (3)	−0.004 (3)
C19	0.026 (2)	0.026 (2)	0.032 (2)	0.0001 (19)	0.0075 (19)	−0.0024 (19)
C20	0.045 (3)	0.039 (3)	0.039 (3)	0.001 (2)	0.013 (2)	0.006 (2)
C21	0.057 (3)	0.028 (3)	0.066 (4)	0.006 (2)	0.019 (3)	0.002 (2)
C22	0.038 (3)	0.043 (3)	0.055 (3)	0.004 (2)	0.013 (3)	−0.014 (2)
C23	0.047 (3)	0.045 (3)	0.044 (3)	0.001 (2)	0.020 (2)	−0.006 (2)
C24	0.048 (3)	0.028 (3)	0.045 (3)	0.000 (2)	0.020 (2)	0.001 (2)

### tert-Butyl [(4-fluoro-3-isopropoxyisoxazol-5-yl)methyl](phenylsulfonyl)carbamate Geometric parameters (Å, º)

**Table:**

S18—O18	1.430 (3)	C9—H9C	0.9800
S18—O19	1.433 (3)	C10—H10A	0.9900
S18—N11	1.672 (4)	C10—H10B	0.9900
S18—C19	1.753 (4)	C14—C15	1.513 (7)
F4—C4	1.333 (5)	C14—C16	1.509 (7)
O1—N2	1.425 (5)	C14—C17	1.530 (7)
O1—C5	1.360 (5)	C15—H15A	0.9800
O6—C3	1.325 (5)	C15—H15B	0.9800
O6—C7	1.478 (5)	C15—H15C	0.9800
O12—C12	1.201 (6)	C16—H16A	0.9800
O13—C12	1.322 (5)	C16—H16B	0.9800
O13—C14	1.492 (5)	C16—H16C	0.9800
N2—C3	1.304 (6)	C17—H17A	0.9800
N11—C10	1.471 (6)	C17—H17B	0.9800
N11—C12	1.394 (6)	C17—H17C	0.9800
C3—C4	1.429 (6)	C19—C20	1.386 (6)
C4—C5	1.340 (6)	C19—C24	1.379 (6)
C5—C10	1.497 (6)	C20—H20	0.9500
C7—H7	1.0000	C20—C21	1.384 (7)
C7—C8	1.505 (7)	C21—H21	0.9500
C7—C9	1.509 (7)	C21—C22	1.379 (7)
C8—H8A	0.9800	C22—H22	0.9500
C8—H8B	0.9800	C22—C23	1.378 (7)
C8—H8C	0.9800	C23—H23	0.9500
C9—H9A	0.9800	C23—C24	1.380 (7)
C9—H9B	0.9800	C24—H24	0.9500
			
O18—S18—O19	119.20 (19)	O12—C12—O13	127.2 (4)
O18—S18—N11	103.28 (19)	O12—C12—N11	122.1 (4)
O18—S18—C19	109.01 (19)	O13—C12—N11	110.7 (4)
O19—S18—N11	109.52 (19)	O13—C14—C15	101.9 (4)
O19—S18—C19	109.01 (19)	O13—C14—C16	109.5 (4)
N11—S18—C19	105.97 (19)	O13—C14—C17	108.6 (4)
C5—O1—N2	109.1 (3)	C15—C14—C17	111.7 (4)
C3—O6—C7	117.1 (3)	C16—C14—C15	112.0 (4)
C12—O13—C14	121.1 (3)	C16—C14—C17	112.6 (4)
C3—N2—O1	105.1 (3)	C14—C15—H15A	109.5
C10—N11—S18	119.1 (3)	C14—C15—H15B	109.5
C12—N11—S18	124.3 (3)	C14—C15—H15C	109.5
C12—N11—C10	116.6 (4)	H15A—C15—H15B	109.5
O6—C3—C4	123.1 (4)	H15A—C15—H15C	109.5
N2—C3—O6	125.7 (4)	H15B—C15—H15C	109.5
N2—C3—C4	111.2 (4)	C14—C16—H16A	109.5
F4—C4—C3	125.3 (4)	C14—C16—H16B	109.5
F4—C4—C5	128.8 (4)	C14—C16—H16C	109.5
C5—C4—C3	105.9 (4)	H16A—C16—H16B	109.5
O1—C5—C10	114.5 (4)	H16A—C16—H16C	109.5
C4—C5—O1	108.6 (4)	H16B—C16—H16C	109.5
C4—C5—C10	136.9 (4)	C14—C17—H17A	109.5
O6—C7—H7	109.5	C14—C17—H17B	109.5
O6—C7—C8	110.2 (4)	C14—C17—H17C	109.5
O6—C7—C9	104.5 (4)	H17A—C17—H17B	109.5
C8—C7—H7	109.5	H17A—C17—H17C	109.5
C8—C7—C9	113.3 (5)	H17B—C17—H17C	109.5
C9—C7—H7	109.5	C20—C19—S18	119.8 (3)
C7—C8—H8A	109.5	C24—C19—S18	118.8 (3)
C7—C8—H8B	109.5	C24—C19—C20	121.4 (4)
C7—C8—H8C	109.5	C19—C20—H20	120.7
H8A—C8—H8B	109.5	C21—C20—C19	118.7 (4)
H8A—C8—H8C	109.5	C21—C20—H20	120.7
H8B—C8—H8C	109.5	C20—C21—H21	120.0
C7—C9—H9A	109.5	C22—C21—C20	120.1 (5)
C7—C9—H9B	109.5	C22—C21—H21	120.0
C7—C9—H9C	109.5	C21—C22—H22	119.7
H9A—C9—H9B	109.5	C23—C22—C21	120.7 (5)
H9A—C9—H9C	109.5	C23—C22—H22	119.7
H9B—C9—H9C	109.5	C22—C23—H23	120.1
N11—C10—C5	111.8 (4)	C22—C23—C24	119.9 (5)
N11—C10—H10A	109.3	C24—C23—H23	120.1
N11—C10—H10B	109.3	C19—C24—C23	119.3 (4)
C5—C10—H10A	109.3	C19—C24—H24	120.4
C5—C10—H10B	109.3	C23—C24—H24	120.4
H10A—C10—H10B	107.9		
			
S18—N11—C10—C5	85.5 (4)	N11—S18—C19—C24	−67.2 (4)
S18—N11—C12—O12	−173.8 (4)	C3—O6—C7—C8	75.5 (5)
S18—N11—C12—O13	7.9 (5)	C3—O6—C7—C9	−162.4 (4)
S18—C19—C20—C21	177.4 (4)	C3—C4—C5—O1	0.9 (5)
S18—C19—C24—C23	−177.9 (4)	C3—C4—C5—C10	−179.7 (5)
F4—C4—C5—O1	−177.5 (4)	C4—C5—C10—N11	4.3 (8)
F4—C4—C5—C10	1.9 (9)	C5—O1—N2—C3	1.3 (5)
O1—N2—C3—O6	179.8 (4)	C7—O6—C3—N2	−5.4 (6)
O1—N2—C3—C4	−0.7 (5)	C7—O6—C3—C4	175.2 (4)
O1—C5—C10—N11	−176.3 (4)	C10—N11—C12—O12	5.0 (6)
O6—C3—C4—F4	−2.1 (7)	C10—N11—C12—O13	−173.3 (4)
O6—C3—C4—C5	179.4 (4)	C12—O13—C14—C15	178.6 (4)
O18—S18—N11—C10	−3.4 (4)	C12—O13—C14—C16	−62.7 (5)
O18—S18—N11—C12	175.4 (3)	C12—O13—C14—C17	60.6 (5)
O18—S18—C19—C20	−134.2 (4)	C12—N11—C10—C5	−93.4 (4)
O18—S18—C19—C24	43.3 (4)	C14—O13—C12—O12	5.0 (7)
O19—S18—N11—C10	−131.4 (3)	C14—O13—C12—N11	−176.7 (4)
O19—S18—N11—C12	47.4 (4)	C19—S18—N11—C10	111.2 (3)
O19—S18—C19—C20	−2.6 (4)	C19—S18—N11—C12	−70.0 (4)
O19—S18—C19—C24	175.0 (4)	C19—C20—C21—C22	0.7 (7)
N2—O1—C5—C4	−1.4 (5)	C20—C19—C24—C23	−0.4 (7)
N2—O1—C5—C10	179.0 (4)	C20—C21—C22—C23	−0.8 (8)
N2—C3—C4—F4	178.4 (4)	C21—C22—C23—C24	0.3 (8)
N2—C3—C4—C5	−0.1 (5)	C22—C23—C24—C19	0.3 (7)
N11—S18—C19—C20	115.2 (4)	C24—C19—C20—C21	−0.1 (7)

### tert-Butyl [(4-fluoro-3-isopropoxyisoxazol-5-yl)methyl](phenylsulfonyl)carbamate Hydrogen-bond geometry (Å, º)

**Table:**

*D*—H···*A*	*D*—H	H···*A*	*D*···*A*	*D*—H···*A*
C10—H10*A*···O19^i^	0.99	2.47	3.121 (6)	123
C15—H15*B*···O18^ii^	0.98	2.59	3.571 (6)	176
C22—H22···O12^iii^	0.95	2.51	3.335 (6)	145
C24—H24···O19^i^	0.95	2.51	3.366 (5)	150

Symmetry codes: (i) *x*, −*y*+1, *z*+1/2; (ii) *x*+1/2, *y*+1/2, *z*; (iii) *x*−1/2, *y*+1/2, *z*.
